# Drug‐Specific Associations of Angiotensin Receptor Blockers With Parkinson's Disease Risk in a Multicenter Electronic Health Record Cohort

**DOI:** 10.1111/fcp.70113

**Published:** 2026-10-18

**Authors:** Kyle Tran, Jichen Yang, Sally Pham, Dylan Luong, Benjamin Andrew Martin, Stefanie Sasaki, Joshua Hahn, Richard Luu, Gha‐hyun Jeffrey Kim

**Affiliations:** ^1^ Department of Clinical Pharmacy Practice, School of Pharmacy and Pharmaceutical Sciences University of California‐Irvine Irvine California USA; ^2^ Robert A. Mah Molecular Innovation Center University of California‐Irvine Irvine California USA; ^3^ Department of Neurobiology and Behavior, Charlie Dunlop School of Biological Sciences University of California Irvine California USA

**Keywords:** angiotensin receptor blockers, drug repositioning, irbesartan, olmesartan, Parkinson's disease, pharmacoepidemiology

## Abstract

**Background:**

Angiotensin receptor blockers (ARBs) have demonstrated neuroprotective effects in preclinical models of Parkinson's disease (PD), but individual ARBs differ in pharmacological properties that may influence their neuroprotective potential.

**Objectives:**

Using large‐scale hospital electronic health records of the University of California health system, we tested whether specific ARBs are associated with lower PD risk.

**Methods:**

We conducted a new‐user, active‐comparator cohort study in the University of California Health Data Warehouse, encompassing 890 712 hypertensive patients across six academic medical centers from 2012 to 2025. Cox proportional hazards models estimated hazard ratios for individual ARB associations with incident PD, adjusting for demographics, nine preindex comorbidities, and index date. Robustness was assessed through propensity score matching and negative‐control analysis.

**Results:**

Drug‐specific Cox analysis identified irbesartan (HR = 0.588, 95% CI 0.434 to 0.796, *p* = 0.0006) and olmesartan (HR = 0.797, 95% CI 0.665 to 0.956, *p* = 0.015) as associated with lower PD risk. Irbesartan survived Bonferroni correction for multiple comparisons, and propensity score matching supported both associations.

**Conclusion:**

In this large multicenter EHR cohort, irbesartan and olmesartan were associated with lower incident Parkinson's disease risk. These findings support further investigation of the renin‐angiotensin system as a therapeutic target in Parkinson's disease and may inform the development or repurposing of agents with favorable central nervous system penetration and neuroprotective potential.

**Abbreviations**
ACEi: angiotensin‐converting enzyme inhibitors
AGTR1: angiotensin II type 1 receptor
ARB: angiotensin receptor blocker
BBB: blood–brain barrier
CI: confidence interval
CNS: central nervous system
EHR: electronic health record
HR: hazard ratio
OMOP: Observational Medical Outcomes Partnership
PD: Parkinson's disease
PPAR‐γ: peroxisome proliferator‐activated receptor gamma
STROBE: strengthening the reporting of observational studies in epidemiology
UCHDW: University of California Health Data Warehouse

## 1 Introduction

Parkinson's disease (PD) is the second most common neurodegenerative disorder worldwide [1, 2] and a rapidly growing cause of disability in aging populations [3, 4, 5]. Despite major advances in symptomatic management, no therapy has yet been established that reproducibly slows or prevents neurodegeneration in PD [6, 7, 8]. This therapeutic gap has increased interest in drug repurposing, particularly for chronic medications with well‐characterized safety profiles, established clinical use, and pharmacologic properties that may be relevant to neuroprotection. This strategy is especially attractive in PD, where long disease latency and slow progression make de novo therapeutic development particularly costly and time‐consuming.

Among candidate pathways for repurposing, the brain renin‐angiotensin system has emerged as a biologically plausible target in PD [6]. Angiotensin II signaling through the angiotensin II type 1 receptor (AGTR1) has been implicated in microglial activation, oxidative stress, mitochondrial dysfunction, and proinflammatory signaling within vulnerable nigrostriatal circuits [9, 10]. In experimental models, inhibition of this pathway has been associated with reduced neuroinflammation and improved dopaminergic neuron survival [11, 12, 13]. These observations have generated interest in angiotensin receptor blockers (ARBs) as possible neuroprotective agents beyond their established role in cardiovascular disease. Interest in this pathway is not confined to PD. An extensive literature in Alzheimer's disease reports that renin‐angiotensin‐acting antihypertensives, and ARBs in particular, are associated with slower cognitive decline and lower dementia incidence, with several analyses indicating greater benefit for agents able to cross the blood–brain barrier than for those that cannot [14, 15]. That broadly similar patterns emerge across two mechanistically distinct neurodegenerative diseases strengthens the rationale for evaluating renin‐angiotensin blockade in PD and argues for attention to differences among individual agents rather than to the class as a whole.

Although individual ARBs can be considered therapeutically interchangeable for certain situations, particularly cardiovascular indications, such as blood pressure reduction, their structural and pharmacological differences may have meaningful consequences for central nervous system effects. In the context of neurodegeneration, ARBs should not be viewed as a uniform class, as properties such as blood–brain barrier penetration, receptor binding kinetics, and off‐target receptor activity vary substantially across individual agents. Although all members of the class antagonize AGTR1 [16, 17, 18], individual ARBs differ in properties that may influence central nervous system effects, including blood–brain barrier penetration [14], receptor binding kinetics [19, 20, 21], tissue distribution, duration of action [22, 23, 24], and peroxisome proliferator‐activated receptor gamma (PPAR‐γ) activity [25, 26]. These differences raise the possibility that protective associations, if present, may be specific to certain agents rather than uniform across the class. This may help explain why prior epidemiologic studies of ARBs and PD have produced inconsistent findings [27, 28].

Real‐world pharmacoepidemiologic data offer an opportunity to identify such drug‐specific signals at scale. Large electronic health record (EHR) datasets can capture routine prescribing patterns across diverse populations and thereby support comparative evaluation of individual agents within established therapeutic classes [29, 30, 31]. In the setting of PD repurposing, this approach may be particularly useful for prioritizing already approved drugs for downstream mechanistic and translational follow‐up.

In this study, we used the University of California Health Data Warehouse, a large multicenter EHR resource, to examine associations between individual ARBs and incident PD in a new‐user, active‐comparator design. We hypothesized that specific ARBs, rather than the class as a whole, would show stronger associations with lower PD risk. Our goal was to identify individual ARBs associated with lower incident PD risk and to prioritize the most promising candidates for follow‐up repurposing studies.

## 2 Materials and Methods

### 2.1 Study Design and Data Source

This was a retrospective, new‐user, active‐comparator cohort study using the University of California Health Data Warehouse (UCHDW) [32, 33]. The UCHDW contains EHR data from six University of California medical centers (UC San Francisco, UC Los Angeles, UC Davis, UC Irvine, UC San Diego, and UC Riverside) encompassing over 11 million patients seen between 2012 and 2025. All data are mapped to the Observational Medical Outcomes Partnership (OMOP) Common Data Model [34, 35]. This study was conducted and is reported in accordance with the STROBE guideline [36, 37]. The overall schematic of the study is shown in Figure 1.

**FIGURE 1:**
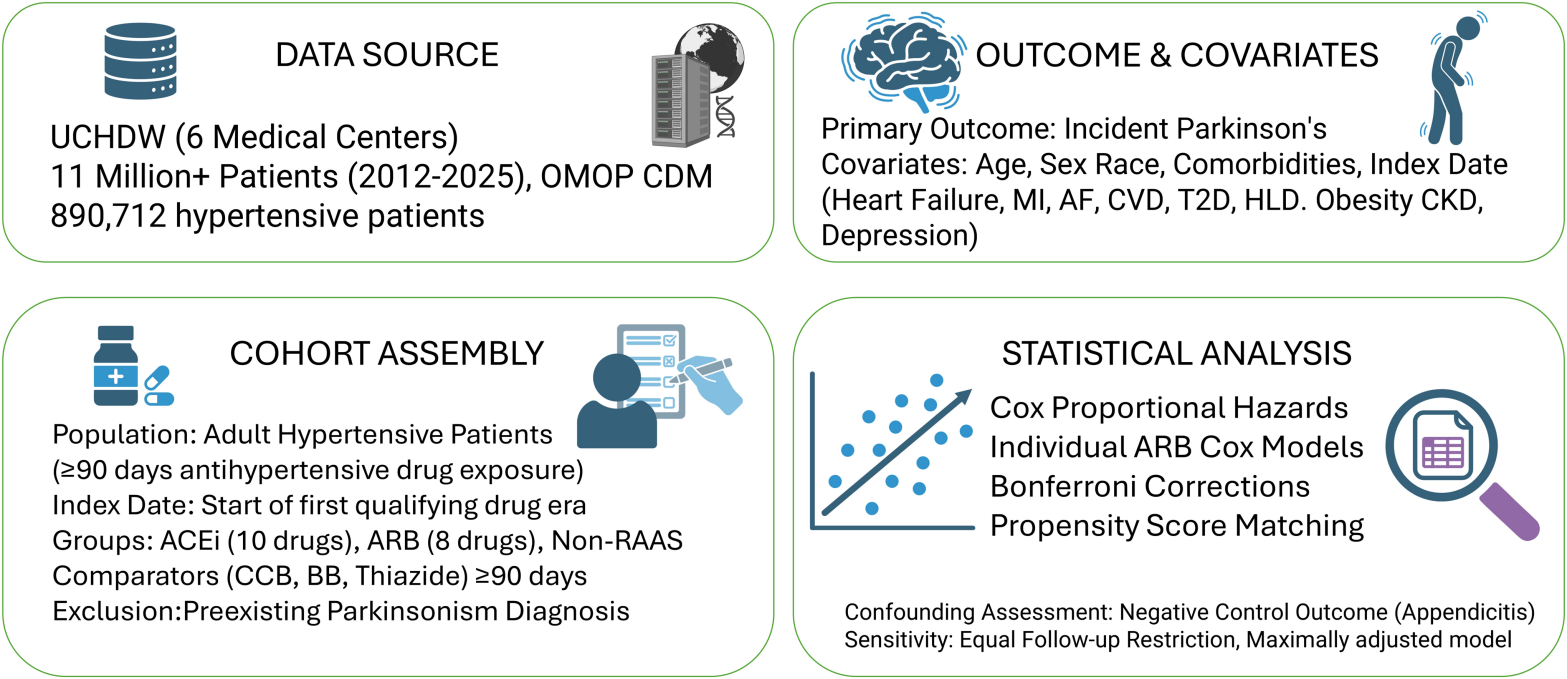
Graphical overview of study design. New‐user, active‐comparator pharmacoepidemiologic study using the University of California Health Data Warehouse (UCHDW), a multicenter electronic health record database of 890 712 hypertensive patients across six academic medical centers mapped to the OMOP Common Data Model.

### 2.2 Cohort Definition

Hypertensive patients with at least one drug era of 90 days or longer for any study antihypertensive were identified and classified by their first qualifying drug era into angiotensin‐converting enzyme inhibitors (ACEi, 10 drugs), ARBs (eight drugs), or non‐RAAS comparators (calcium channel blockers, beta‐blockers, and thiazide diuretics, 20 drugs). The index date was defined as the start of the first qualifying drug era. Patients with any diagnosis of PD before the index date were excluded. Patients with any diagnosis of secondary, drug‐induced, neuroleptic‐induced, or vascular parkinsonism at any time during the observation period were also excluded. The primary outcome was incident PD diagnosed after the index date. The study design and the geographical distribution across the medical centers is represented in Figure 2.

**FIGURE 2:**
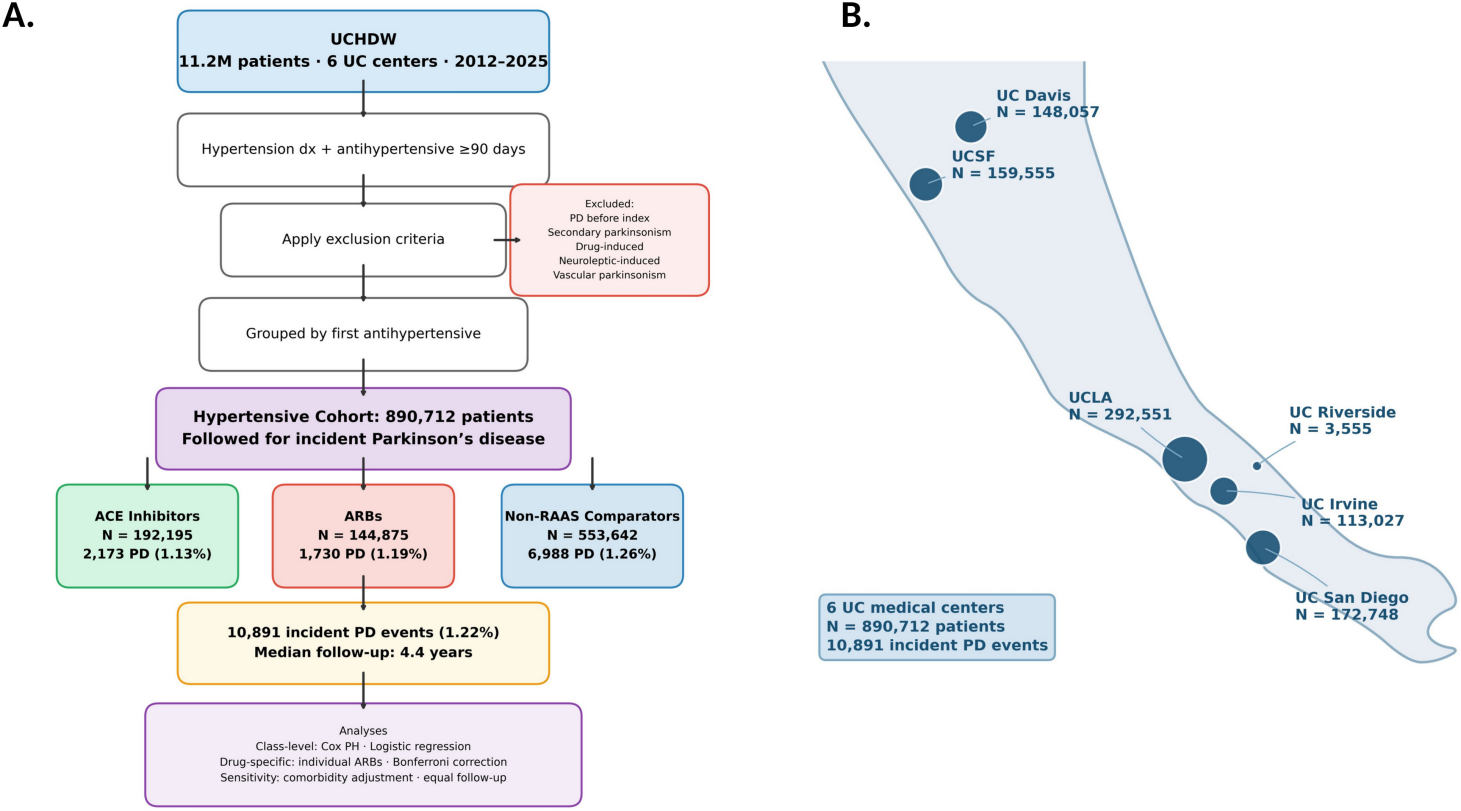
Study design and data source. (A) Cohort assembly flow diagram showing cohort assembly from 11.2 million patients in the University of California Health Data Warehouse (UCHDW). (B) Geographic distribution of patients across six UC medical centers. Circle size is proportional to site contribution.

### 2.3 Covariates

Covariates included age at index date, sex, self‐reported race, ethnicity, index date (to account for secular trends in PD diagnosis and differential entry time across drug groups), and nine preindex comorbidities: heart failure, myocardial infarction, atrial fibrillation, cerebrovascular disease, type 2 diabetes mellitus, chronic kidney disease, depression, hyperlipidemia, and obesity. Comorbidities were ascertained using comprehensive OMOP concept ID sets verified against the Athena vocabulary [38].

### 2.4 Statistical Analysis

The primary analysis used Cox proportional hazards models to estimate hazard ratios (HRs) for the association between antihypertensive drug exposure and incident PD. The active‐comparator design was chosen to reduce confounding by indication and healthcare utilization patterns that could bias comparisons with untreated populations. Because ARB uptake increased over calendar time within the UC system, temporal adjustment was incorporated to reduce bias from differential entry time. Follow‐up time was measured from the index date to incident PD diagnosis or censoring at the last recorded visit in the EHR, whichever occurred first. Drug‐specific analyses tested seven individual ARBs against the non‐RAAS comparator pool. Eprosartan was excluded because only 8 exposed patients were identified. Bonferroni correction for seven comparisons established a significance threshold of alpha = 0.0071. The same drug‐specific model was then applied to the 10 individual ACEi against the identical non‐RAAS comparator pool, using the same covariates, follow‐up definition, and censoring rules, in order to test whether protective associations within the renin‐angiotensin system were confined to ARBs. Bonferroni correction for 10 comparisons established a significance threshold of alpha = 0.005 for that analysis. Propensity score matching (PSM) was performed against the non‐RAAS comparator pool as a sensitivity analysis. Propensity scores were estimated from all baseline covariates including index date, and one‐to‐one nearest‐neighbor matching without replacement used a caliper of 0.2 standard deviations of the propensity score logit. Covariate balance was assessed using standardized mean differences (SMD). Odds ratios were calculated in the matched cohorts to assess associations with incident PD. Additionally, matching was done to draw comparators to treated patients one to one, so the matched estimate is an average treatment effect in the treated, and hazard ratios as well as odds ratios were computed in the matched cohorts. Site was added as a covariate using the six UC medical centers. Individual ARBs were compared head to head against losartan, the most widely prescribed agent in the class, so that the reference group was another ARB rather than the non‐RAAS pool. Analyses were repeated within index year strata of 2012 to 2017 and 2018 to 2025. Because death competes with a PD diagnosis, cumulative incidence functions treating death as a competing event were estimated alongside the cause‐specific hazard models. Finally, the parkinsonism exclusion, which in the primary analysis used diagnoses recorded at any time, was restricted to diagnoses recorded on or before the index date.

### 2.5 Ethics

This study used a limited data set from the University of California Health Data Warehouse, a partially deidentified research data asset mapped to the OMOP Common Data Model. Use of UCHDW data for research is deemed not to constitute human subjects research by the University of California Institutional Review Board and does not require IRB approval, provided that data are stored and analyzed within the secure computing environment. Individual informed consent was not required as the study used only deidentified records.

## 3 Results

### 3.1 Study Cohort

From 11.2 million patients in the UCHDW, 890 712 met inclusion criteria: 192 195 ACEi users, 144 875 ARB users, and 553 642 non‐RAAS comparators. There were 10 891 incident PD events over a median follow‐up of 4.49 years for comparators, 4.69 years for ACEi, and 3.70 years for ARB users (Table 1). ARB users were older (mean 63.1 years) than ACEi users (60.3 years) and comparators (62.4 years) and had a higher proportion of Asian patients (15.0%). The cohort spanned six UC academic medical centers, with UCLA contributing the largest proportion (*N* = 292 551, 32.8%), followed by UCSD (*N* = 172 748, 19.4%), UCSF (*N* = 159 555, 17.9%), UCD (*N* = 148 057, 16.6%), UCI (*N* = 113 027, 12.7%), and UCR (*N* = 3555, 0.4%). Site‐level characteristics are presented in Table S1. Mean index date differed across drug classes: ARB users entered the system later (February 2019) than comparators (December 2017) and ACEi users (September 2017), reflecting differential adoption of ARBs in UC prescribing over time. This 14‐month difference between ARB and comparator entry motivated the inclusion of index date as a covariate in all adjusted models.

**TABLE 1:** Baseline characteristics by antihypertensive drug class.

	ACEi (*N* = 192 195)	ARB (*N* = 144 875)	Comparator (*N* = 553 642)
Demographics			
Age, mean, years	60.3	63.1	62.4
Male (%)	56.5	50.2	48.7
White (%)	58.3	55.1	55.8
Black (%)	5.5	4.1	7.8
Asian (%)	8.9	15.0	11.4
Hispanic (%)	20.1	14.8	15.4
Comorbidities			
Heart failure (%)	3.0	3.5	2.9
Myocardial infarction (%)	0.6	0.6	0.6
Atrial fibrillation (%)	1.5	2.0	2.6
Type 2 diabetes (%)	9.5	7.3	5.4
Chronic kidney disease (%)	2.1	2.4	2.8
Depression (%)	4.1	4.2	4.2
Hyperlipidemia (%)	13.1	14.7	12.1
Obesity (%)	4.6	4.8	4.0
Cerebrovascular disease (%)	1.1	0.9	0.9
Exposure and outcome			
Drug era, mean, days	681	587	668
Median follow‐up (years)	4.69	3.70	4.49
Mean index date	September 2017	February 2019	December 2017
PD events, *n* (%)	2173 (1.13)	1730 (1.19)	6988 (1.26)

### 3.2 Individual ARB Analysis

Among the seven evaluable ARBs, irbesartan and olmesartan emerged as the two leading signals associated with lower incident PD risk (Table 2, Figure 3). Irbesartan (*N* = 4784, 42 PD events) showed the strongest association (HR = 0.588, 95% CI 0.434 to 0.796, *p* = 0.0006), surviving Bonferroni correction for seven comparisons. Olmesartan (*N* = 10 440, 118 PD events) showed a concordant lower PD hazard (HR = 0.797, 95% CI 0.665 to 0.956, *p* = 0.015). Together, these findings nominate irbesartan and olmesartan as the highest‐priority ARB repurposing candidates for follow‐up study.

**TABLE 2:** Individual ARB Associations with Parkinson's disease (Cox proportional hazards).

Drug	*N*	PD	HR	95% CI	*p*
Irbesartan	4784	42	0.588	0.434–0.796	0.0006
Olmesartan	10 440	118	0.797	0.665–0.956	0.015
Telmisartan	4434	52	0.906	0.689–1.190	0.48
Losartan	96 761	1054	1.016	0.951–1.084	0.64
Valsartan	26 408	437	1.015	0.921–1.118	0.77
Candesartan	1526	23	—	—	Low sample size
Azilsartan	514	4	—	—	Low sample size
Eprosartan^a^	8	0	—	—	Excluded

^a^ Eprosartan was excluded from drug‐specific modeling because only 8 exposed patients and no PD events were identified. Candesartan and azilsartan were retained in the table but not modeled because event counts were insufficient for stable estimation.

**FIGURE 3:**
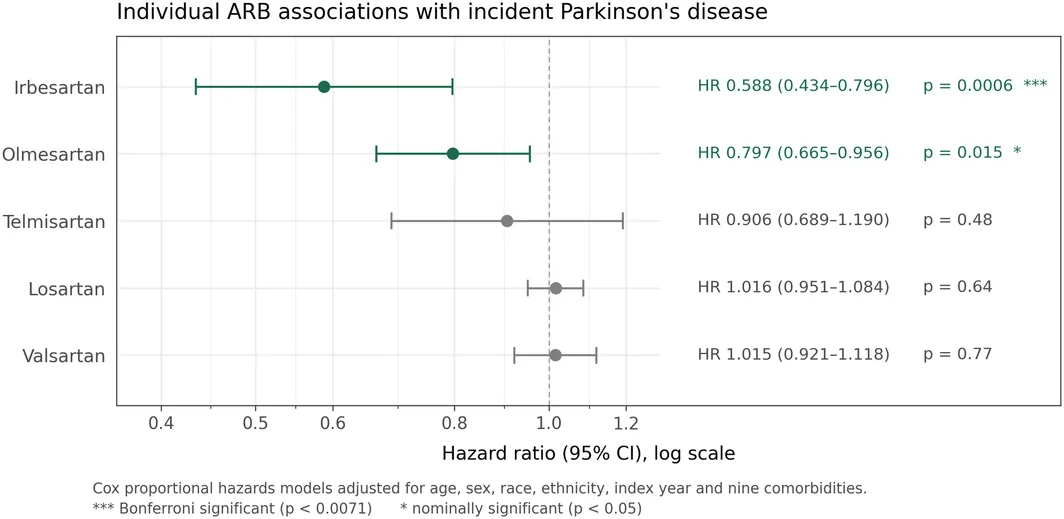
Forest plot of individual ARB hazard ratios for Parkinson's disease. Cox proportional hazards models were adjusted for age, sex, race, ethnicity, index date, and nine comorbidities. Irbesartan and olmesartan showed the strongest protective associations among evaluable individual ARBs. Horizontal bars indicate 95% confidence intervals. The dashed vertical line indicates HR = 1.0. Candesartan and azilsartan were omitted because event counts were insufficient for stable estimation.

Other individual ARBs did not show statistically supported protective associations. Losartan (*N* = 96 761, 1054 PD events, HR = 1.016, 95% CI 0.951 to 1.084, *p* = 0.64) showed no association despite being the largest and best‐powered subgroup, indicating that lack of statistical power does not explain the absence of a protective signal for this drug. Valsartan (*N* = 26 408, 437 PD events, HR = 1.015, 95% CI 0.921 to 1.118, *p* = 0.77) showed no association after adjustment. Valsartan users had substantially higher healthcare utilization than losartan users (mean 403 vs. 299 visits, 54 vs. 44 distinct diagnoses) and higher heart failure prevalence (4.9% vs. 3.4%), consistent with channeling toward sicker patients. The sample size of Candesartan (23 events) and azilsartan (4 events) were too small for informative analysis.

### 3.3 Individual ACE Inhibitor Analysis

To determine whether the drug‐specific pattern observed among ARBs extended to inhibition of the renin‐angiotensin system more broadly, the same Cox model was applied to the 10 individual ACEi against the non‐RAAS comparator pool (Table S3). Considered as a class, ACEi use was not associated with incident PD (HR = 0.958, 95% CI 0.913 to 1.006, *p* = 0.082). At the drug level, none of the seven evaluable agents met the Bonferroni‐corrected threshold of alpha = 0.005, and none reached nominal significance. Lisinopril, the most frequently prescribed ACEi and by far the best‐powered subgroup (*N* = 150 822, 1598 PD events), showed HR = 0.953 (95% CI 0.902 to 1.006, *p* = 0.08); estimates for benazepril, enalapril, ramipril, captopril, quinapril, and fosinopril ranged from HR = 0.822 to 1.497 (all *p* > 0.18), and trandolapril, perindopril, and moexipril had too few events for stable estimation. The absence of a drug‐specific signal among ACEi indicates that the associations observed for irbesartan and olmesartan are not a general property of pharmacological interruption of the renin‐angiotensin system.

### 3.4 Sensitivity Analyses

Propensity score matching supported the Cox findings for both prioritized ARBs. Irbesartan PSM (4784 matched pairs, max SMD = 0.047) yielded OR = 0.580 (95% CI 0.395 to 0.850, *p* = 0.006), essentially identical to the Cox HR of 0.588. The olmesartan matched analysis was computed in the cohort described in Section 3.5 (10 369 matched pairs, max SMD = 0.046), yielding OR = 0.816 and a matched‐cohort HR of 0.764 (95% CI 0.598 to 0.975, *p* = 0.031), consistent with the Cox HR of 0.797. Together, these analyses suggest that the irbesartan and olmesartan signals are reproducible across both the primary time‐to‐event analysis and the matched design. As a negative control, the association between ARB use and incident appendicitis was not significant (OR = 0.914, 95% CI 0.822 to 1.017, *p* = 0.094), suggesting that the observed PD associations are not attributable to a general healthy‐user effect.

### 3.5 Robustness of the Irbesartan and Olmesartan Associations

A series of additional analyses examined the robustness of the irbesartan and olmesartan associations (Table S4). These were run in a cohort rebuilt from source, which recovered the primary estimates closely (irbesartan HR = 0.630, olmesartan HR = 0.820, losartan HR = 0.973), and all values below refer to that cohort. Adding site as a covariate left the estimates essentially unchanged (irbesartan HR = 0.641, 95% CI 0.478 to 0.859, *p* = 0.0029; olmesartan HR = 0.842, 95% CI 0.703 to 1.009, *p* = 0.062). Restricting the parkinsonism exclusion to diagnoses recorded on or before the index date, so that no post‐index information contributed to cohort membership, also preserved both signals (irbesartan HR = 0.669, 95% CI 0.509 to 0.879, *p* = 0.0039; olmesartan HR = 0.784, 95% CI 0.657 to 0.936, *p* = 0.0071). Estimates were directionally consistent across index year strata. Event counts within strata were small for irbesartan. In a head‐to‐head comparison using losartan rather than the non‐RAAS pool as the reference, irbesartan retained a lower PD hazard (HR = 0.637, 95% CI 0.473 to 0.859, *p* = 0.0031) and olmesartan was borderline (HR = 0.829, 95% CI 0.686 to 1.002, *p* = 0.053). In the matched cohorts, which estimate an average treatment effect in the treated, hazard ratios agreed with the odds ratios (irbesartan HR = 0.639, 95% CI 0.437 to 0.935, *p* = 0.021, 4727 pairs, maximum SMD 0.032; olmesartan HR = 0.764, 95% CI 0.598 to 0.975, *p* = 0.031, 10 369 pairs, maximum SMD 0.046). Death occurred before a PD diagnosis in 6.0% of comparators, 4.4% of irbesartan users, and 3.4% of olmesartan users; accounting for death as a competing event, the five‐year cumulative incidence of PD was 1.19% in comparators, 0.99% for irbesartan, 1.08% for olmesartan, and 1.16% for losartan.

## 4 Discussion

In this large multicenter EHR cohort, irbesartan and olmesartan emerged as the two ARBs most strongly associated with lower incident Parkinson's disease risk. The magnitude of the irbesartan association, together with the concordant olmesartan signal, suggests that clinically meaningful within‐class differences may exist among ARBs with respect to PD‐relevant biology. From a repurposing perspective, these findings are important because they identify specific, already approved drugs that can be prioritized for mechanistic follow‐up and translational testing.

Sensitivity analyses strengthened confidence in both lead signals. The translational appeal of these findings lies in the fact that both irbesartan and olmesartan are already established antihypertensives with well‐characterized clinical use. Although observational associations cannot establish disease modification [39, 40, 41], prioritizing approved drugs with known prescribing patterns, tolerability, and real‐world exposure histories may shorten the path from epidemiologic signal detection to experimental validation [42, 43, 44]. In this sense, the present study provides a practical starting point for a focused repurposing pipeline rather than a purely descriptive association screen.

The pharmacological basis underlying the protective associations observed for irbesartan and olmesartan remains to be determined. Individual ARBs differ in multiple properties including BBB penetration, PPAR‐γ agonist activity, receptor binding kinetics, and duration of AT1 blockade. Individual ARBs exhibit a range of reported BBB penetration profiles, and ongoing research continues to refine our understanding of their central nervous system access. Prior preclinical, pharmacokinetic, and clinical studies have yielded differing conclusions regarding which ARBs meaningfully access the CNS. No single pharmacological characteristic cleanly distinguishes the protective from nonprotective ARBs in this analysis. Irbesartan has been reported to exhibit moderate BBB penetration and PPAR‐γ agonist activity with neuroprotective potential in animal models of inflammation [45, 46]. Olmesartan has shown neuroprotective activity in animal models of dopaminergic neurodegeneration [47, 48, 49] despite limited BBB penetration in pharmacokinetic studies.

Beyond blood–brain barrier penetration and PPAR‐γ activity, the two agents identified here share features of receptor engagement that distinguish them from losartan. Irbesartan and olmesartan both produce insurmountable, slowly reversible AT1 blockade with prolonged receptor residence times, whereas losartan binds in a surmountable, rapidly dissociating manner [19, 50]. Structurally, this behavior has been attributed to substituents on the heterocyclic ring that form additional contacts within the receptor binding pocket, including the carboxyl and hydroxyisopropyl groups of olmesartan and the spirocyclopentyl imidazolinone of irbesartan, which engage residues that losartan and the acyclic derivative valsartan do not [20, 21]. Several tightly binding ARBs additionally behave as inverse agonists at AT1, suppressing constitutive receptor activity rather than only displacing angiotensin II [17]. Whether prolonged occupancy or inverse agonism is relevant to dopaminergic neuroprotection has not been tested directly. Candesartan and telmisartan share insurmountable binding yet showed no protective association in this cohort, although both were limited by small event counts, so receptor residence time alone does not account for the pattern observed here. These structural considerations are therefore best regarded as hypotheses for controlled preclinical comparison rather than as an explanation of the present findings.

These findings may help explain the divergent results observed across prior epidemiologic studies of ARBs and PD. Several large registry studies and meta‐analyses have reported protective associations for ARBs as a class, whereas others found no significant effect [27, 28]. Our results suggest that these discrepancies may reflect differences in the relative prescribing proportions of individual ARBs across healthcare systems and study populations. Future pharmacoepidemiologic studies should account for the specific ARB composition of their study populations when interpreting class‐level results.

These results also bear on how much explanatory weight the angiotensin II hypothesis for PD can currently carry. If reduced angiotensin II signaling were by itself sufficient to lower PD risk, protective associations should have been distributed broadly across agents that interrupt the pathway. That is not what we observed. Losartan, the largest and best‐powered ARB subgroup, showed no association, and no individual ACE inhibitor showed a protective association, with the class‐level estimate likewise null; the associations were instead concentrated in two agents. That neither the ACE inhibitor class nor any individual ACE inhibitor showed protection is informative, since these agents interrupt the same pathway upstream. We therefore read these results as evidence that inhibition of angiotensin II signaling is not on its own a sufficient account of the effect, rather than as evidence against the relevance of angiotensin II. The preclinical work implicating brain angiotensin II in microglial activation and dopaminergic vulnerability remains well supported [9, 10, 12, 13]. What these data suggest is that pathway inhibition adequate for systemic blood pressure control does not reliably produce the central receptor engagement that such a mechanism would require. Distinguishing between these possibilities will require agent‐level rather than class‐level evaluation, in experimental models as well as in epidemiology.

This study shows that evaluating individual agents within established therapeutic classes may help identify actionable neuroprotective candidates. The consistency of results across time‐aware and matched analyses increases confidence that these associations are not explained solely by differential follow‐up or measured baseline differences.

### 4.1 Limitations

The UCHDW is restricted to California academic medical centers, which may limit generalizability to other healthcare settings and prescribing environments. We also lacked detailed information on medication dose, adherence, and treatment changes over time, which may have introduced variation in exposure. Furthermore, comorbidity ascertainment was restricted to diagnoses captured within the UCHDW, which begins in 2012, potentially underestimating baseline disease burden for patients entering early in the observation window. Smoking status, socioeconomic position, and educational attainment are not reliably recorded in the UCHDW and could not be included as covariates, and each is plausibly associated with both antihypertensive selection and PD ascertainment. Mortality also differed across exposure groups, with 6.0% of comparators dying before any PD diagnosis compared with 4.4% of irbesartan and 3.4% of olmesartan users, indicating that the comparator pool was on average less healthy; because earlier death reduces the opportunity to receive a PD diagnosis, this pattern would tend to bias the drug associations toward the null rather than produce them. Finally, residual confounding [51, 52] from unmeasured lifestyle, prodromal, or disease‐severity factors cannot be excluded despite the active‐comparator design, temporal adjustment, matching, and negative‐control analysis.

## 5 Conclusion

Irbesartan and olmesartan were associated with lower incident Parkinson's disease risk in this multicenter EHR cohort. Across primary and sensitivity analyses, both emerged as leading ARB repurposing candidates, with irbesartan showing the most consistent signal across analytical approaches. These findings support focused preclinical validation and subsequent translational evaluation of both drugs in Parkinson's disease. More broadly, they underscore the continued value of systematic drug repurposing and candidate discovery efforts for Parkinson's disease, where disease‐modifying therapies remain urgently needed.

## Author Contributions

**Gha‐hyun Jeffrey Kim:** conceptualization, methodology, software, formal analysis, investigation, data curation, writing, visualization. **Kyle Tran, Jichen Yang:** methodology, investigation, formal analysis. **Sally Pham, Dylan Luong, Benjamin Andrew Martin, Stefanie Sasaki, Joshua Hahn, Richard Luu:** data curation, literature review.

## Funding

GJK was supported by the National Institutes of Health AIM‐AHEAD fellowship program.

## Ethics Statement

This study used a limited, deidentified data set from the University of California Health Data Warehouse. Under University of California Institutional Review Board policy, use of these data for research does not constitute human subjects research and therefore did not require IRB approval or individual informed consent.

## Consent

The authors have nothing to report.

## Conflicts of Interest

The authors declare no conflicts of interest.

## Supporting information


**Table S1:** Site‐level characteristics and ARB–Parkinson's disease associations across UC medical centers.
**Table S2:** OMOP concept IDs used for cohort definition.
**Table S3:** Individual ACE inhibitor associations with Parkinson's disease (Cox proportional hazards).
**Table S4:** Sensitivity and robustness analyses for irbesartan and olmesartan.

## Data Availability

UCHDW data are available to University of California researchers with appropriate data use agreements through the Center for Data‐driven Insights and Innovation at UC Health. Analysis code or other helpful tools can be shared upon request to the author.
