# Supplementary material for: Drug‐Specific Associations of Angiotensin Receptor Blockers With Parkinson's Disease Risk in a Multicenter Electronic Health Record Cohort
Source: Fundam Clin Pharmacol. 2026 Oct 18;40(6):e70113. doi: 10.1111/fcp.70113 (PMC13677279; doi:10.1111/fcp.70113)
Supplement: Supplementary file 1 [file FCP-40-0-s001.docx]

**Supplementary Materials**

**Table S1. Site-Level Characteristics and ARB–Parkinson’s Disease Associations Across UC Medical Centers**

| **Site** | **N** | **PD** | **Age** | **PD %** |
| --- | --- | --- | --- | --- |
| UCLA | 292,551 | 3,253 | 62.0 | 1.11 |
| UCSD | 172,748 | 2,336 | 62.3 | 1.35 |
| UCSF | 159,555 | 2,040 | 62.4 | 1.28 |
| UCD | 148,057 | 1,727 | 60.8 | 1.17 |
| UCI | 113,027 | 1,498 | 63.1 | 1.33 |
| UCR | 3,555 | 33 | 57.6 | 0.93 |
| Unassigned | 1,219 | 4 | — | 0.33 |
| **Pooled** | **890,712** | **10,891** | **62.1** | **1.22** |

**Table S2. OMOP Concept IDs Used for Cohort Definition**

| **Category** | **Drugs/Conditions** | **OMOP Concept IDs** |
| --- | --- | --- |
| **ARBs (8)** | losartan, olmesartan, irbesartan, telmisartan, candesartan, azilsartan, eprosartan, valsartan | 1367500, 40226742, 1347384, 1317640, 1351557, 40235485, 1346686, 1308842 |
| **ACEi (10)** | lisinopril, benazepril, enalapril, ramipril, captopril, quinapril, fosinopril, trandolapril, perindopril, moexipril | 1308216, 1335471, 1341927, 1334456, 1340128, 1331235, 1363749, 1342439, 1373225, 1310756 |
| **CCB (6)** | amlodipine, diltiazem, nifedipine, nicardipine, felodipine, verapamil | 1332418, 1328165, 1318853, 1318137, 1353776, 1307863 |
| **BB (11)** | atenolol, nebivolol, bisoprolol, nadolol, timolol, pindolol, acebutolol, carvedilol, metoprolol, labetalol, propranolol | 1314002, 1314577, 1338005, 1313200, 902427, 1345858, 1319998, 1346823, 1307046, 1386957, 1353766 |
| **Thiazide (3)** | hydrochlorothiazide, chlorthalidone, indapamide | 974166, 1395058, 978555 |
| **Hypertension (4)** | Essential HTN, hypertensive disorder, benign HTN, benign essential HTN | 320128, 316866, 4028741, 312648 |
| **Parkinson’s disease (1)** | Parkinson’s disease | 381270 |
| **Parkinsonism exclusion (4)** | Secondary, drug-induced, neuroleptic-induced, vascular parkinsonism | 374013, 4171569, 4248716, 4046093 |
| **Heart failure (7)** | Congestive HF, HF, left HF, right HF, HF with reduced EF, chronic systolic HF, chronic diastolic HF | 316139, 319835, 4059290, 321319, 40479192, 4229440, 4233424 |
| **Myocardial infarction (5)** | Acute MI, old MI, subendocardial MI, acute ST segment elevation MI, silent MI | 4270024, 312327, 434376, 438170, 4329847 |
| **Atrial fibrillation (3)** | Atrial fibrillation, chronic AF, paroxysmal AF | 313217, 314665, 4141360 |
| **Cerebrovascular disease (6)** | Cerebral infarction, cerebrovascular accident, occlusion of cerebral artery, cerebral embolism, cerebral thrombosis, cerebral atherosclerosis | 443454, 439847, 4043731, 372924, 441874, 375557 |
| **Type 2 diabetes (4)** | DM2 without complication, DM2, DM2 with renal complication, DM2 with ophthalmic complication | 4193704, 201820, 4099651, 4063043 |
| **Chronic kidney disease (6)** | CKD, CKD stage 3, CKD stage 4, CKD stage 5, CKD stage 3a, CKD stage 3b | 46271022, 443601, 192671, 443612, 443611, 443614 |
| **Depression (5)** | Depressive disorder, major depressive disorder, recurrent major depression, dysthymia, depressive episode | 440383, 4152280, 4049623, 4282096, 436670 |
| **Hyperlipidemia (3)** | Hyperlipidemia, pure hypercholesterolemia, mixed hyperlipidemia | 432867, 437833, 4145825 |
| **Obesity (1)** | Obesity | 433736 |
| **Negative control (3)** | Acute appendicitis, appendicitis, perforated appendicitis | 440448, 441589, 4265600 |

**Table S3. Individual ACE Inhibitor Associations with Parkinson’s Disease (Cox Proportional Hazards)**

| **Drug** | **N** | **PD** | **HR** | **95% CI** | **p** |
| --- | --- | --- | --- | --- | --- |
| Lisinopril | 150,822 | 1,598 | 0.953 | 0.902–1.006 | 0.08 |
| Benazepril | 21,191 | 295 | 0.958 | 0.853–1.077 | 0.48 |
| Enalapril | 6,222 | 93 | 1.004 | 0.818–1.232 | 0.97 |
| Ramipril | 5,898 | 117 | 1.038 | 0.864–1.246 | 0.69 |
| Captopril | 528 | 11 | 1.497 | 0.829–2.705 | 0.18 |
| Quinapril | 2,555 | 37 | 0.823 | 0.595–1.137 | 0.24 |
| Fosinopril | 790 | 13 | 0.822 | 0.477–1.416 | 0.48 |
| Trandolapril | 101 | 3 | — | — | low sample size |
| Perindopril | 103 | 1 | — | — | low sample size |
| Moexipril | 64 | 1 | — | — | low sample size |

Cox proportional hazards models adjusted for age, sex, race, ethnicity, index date, and nine comorbidities, with the non-RAAS comparator pool as reference. Bonferroni-corrected threshold for ten comparisons, alpha = 0.005. Class-level ACEi versus comparator: HR = 0.958 (95% CI 0.913 to 1.006, p = 0.082).

**Table S4. Sensitivity and Robustness Analyses for Irbesartan and Olmesartan**

| **Analysis** | **Irbesartan HR (95% CI)** | **p** | **Olmesartan HR (95% CI)** | **p** | **Losartan HR** |
| --- | --- | --- | --- | --- | --- |
| Primary, rebuilt cohort | 0.630 (0.470–0.845) | 0.0020 | 0.820 (0.684–0.982) | 0.031 | 0.973 |
| Site-adjusted | 0.641 (0.478–0.859) | 0.0029 | 0.842 (0.703–1.009) | 0.062 | 0.977 |
| Parkinsonism exclusion pre-index only | 0.669 (0.509–0.879) | 0.0039 | 0.784 (0.657–0.936) | 0.0071 | 0.972 |
| Index year 2012–2017 | 0.542 (0.346–0.851) | 0.0078 | 0.841 (0.664–1.064) | 0.15 | 1.012 |
| Index year 2018–2025 | 0.722 (0.491–1.063) | 0.099 | 0.801 (0.606–1.060) | 0.12 | 0.939 |
| Head-to-head vs losartan | 0.637 (0.473–0.859) | 0.0031 | 0.829 (0.686–1.002) | 0.053 | — |
| Matched cohort (ATT), HR | 0.639 (0.437–0.935) | 0.021 | 0.764 (0.598–0.975) | 0.031 | — |

Cox proportional hazards models adjusted for age, sex, race, ethnicity, index date, and nine comorbidities unless otherwise stated, with the non-RAAS comparator pool as reference except in the head-to-head row. Index year strata omit index year as a covariate. ATT, average treatment effect in the treated. SMD, standardized mean difference.
